# Enhanced Osteogenic and Vasculogenic Differentiation Potential of Human Adipose Stem Cells on Biphasic Calcium Phosphate Scaffolds in Fibrin Gels

**DOI:** 10.1155/2016/1934270

**Published:** 2016-07-28

**Authors:** Fransisca A. S. van Esterik, Behrouz Zandieh-Doulabi, Cornelis J. Kleverlaan, Jenneke Klein-Nulend

**Affiliations:** ^1^Department of Oral Cell Biology, Academic Centre for Dentistry Amsterdam (ACTA), University of Amsterdam and Vrije Universiteit Amsterdam, MOVE Research Institute Amsterdam, Amsterdam, Netherlands; ^2^Department of Dental Materials Science, Academic Centre for Dentistry Amsterdam (ACTA), University of Amsterdam and Vrije Universiteit Amsterdam, MOVE Research Institute Amsterdam, Amsterdam, Netherlands

## Abstract

For bone tissue engineering synthetic biphasic calcium phosphate (BCP) with a hydroxyapatite/*β*-tricalcium phosphate (HA/*β*-TCP) ratio of 60/40 (BCP60/40) is successfully clinically applied, but the high percentage of HA may hamper efficient scaffold remodelling. Whether BCP with a lower HA/*β*-TCP ratio (BCP20/80) is more desirable is still unclear. Vascular development is needed before osteogenesis can occur. We aimed to test the osteogenic and/or vasculogenic differentiation potential as well as degradation of composites consisting of human adipose stem cells (ASCs) seeded on BCP60/40 or BCP20/80 incorporated in fibrin gels that trigger neovascularization for bone regeneration. ASC attachment to BCP60/40 and BCP20/80 within 30 min was similar (>93%). After 11 days of culture BCP20/80-based composites showed increased alkaline phosphatase activity and* DMP1* gene expression, but not* RUNX2* and osteonectin expression, compared to BCP60/40-based composites. BCP20/80-based composites also showed enhanced expression of the vasculogenic markers* CD31* and* VEGF189*, but not* VEGF165* and endothelin-1. Collagen-1 and collagen-3 expression was similar in both composites. Fibrin degradation was increased in BCP20/80-based composites at day 7. In conclusion, BCP20/80-based composites showed enhanced osteogenic and vasculogenic differentiation potential compared to BCP60/40-based composites* in vitro*, suggesting that BCP20/80-based composites might be more promising for* in vivo* bone augmentation than BCP60/40-based composites.

## 1. Introduction

Bone tissue engineering has become a promising alternative for bone reconstruction. It is based on combinations of scaffolds, (stem) cells, and mechanical and/or chemical stimuli [1]. Autologous bone is the golden standard for clinical bone augmentation, for example, maxillary sinus floor elevation (MSFE) [2]. An alternative to the golden standard is biphasic calcium phosphate (BCP). BCPs are used as bone substitute materials for dental and orthopaedic applications. Ellinger et al. (1986) were the first to use the term BCP to describe bioceramics composed of hydroxyapatite (HA) and *β*-tricalcium phosphate (*β*-TCP) [3]. The chemical composition of BCP resembles the inorganic part of the natural bone matrix [4]. LeGeros and Daculsi reported in 1986 the first basic studies on the preparation of BCP and its* in vitro *properties [5, 6]. Thereafter, there was a significant increase in manufacture and use of commercial BCP as bone substitute. HA is rigid, brittle, and hardly resorbed after application in, for example, MSFE, while *β*-TCP degrades faster and has a different resorption pattern [7]. For efficient scaffold remodelling, a BCP with an optimum ratio of HA and *β*-TCP is desired. BCP with a HA/*β*-TCP ratio of 60/40 (BCP60/40) is successfully applied clinically [4, 8, 9], but the high percentage of HA may hamper efficient scaffold remodelling. Whether BCP with a HA/*β*-TCP ratio of 20/80 (BCP20/80) is more desirable compared to BCP60/40 is still unclear.

BCP supports attachment, proliferation, and osteogenic differentiation of progenitor cells, and can be combined with regeneration-competent stem cells, such as adipose stem cells (ASCs), to introduce osteogenic bioactivity [10, 11]. Clinically relevant stem cell numbers with a high proliferative capacity can be easily extracted from human adipose tissue [12, 13]. ASCs have multilineage potential including the osteogenic lineage [14], and probably the endothelial lineage [15]. In the field of bone tissue engineering, ASCs have been successfully used for bone augmentation in MSFE [16].

Adequate vascularization is pivotal for cell survival of transplanted regeneration-competent stem cells in cell-based bone constructs. Osteogenesis and vasculogenesis are tightly coupled processes, and vascular development needs to be induced before osteogenesis can take place. This complexity has been a major challenge for engineering viable and functional bone grafts [17, 18]. Bioscaffolds, like natural human-derived extracellular matrix scaffolds, enhance vascular development [19]. Another bioscaffold is fibrin, an insoluble, elastic protein playing a crucial role in blood clotting and wound healing. Cell-based bone constructs might be combined with bioscaffolds to induce vascular development.

BCP combined with bioscaffolds, like fibrin, has demonstrated its ability to fill bone defects and promote bone healing in animal and clinical studies [20–23]. Fibrin gel provides a biocompatible carrier for cellular function, survival, proliferation, and differentiation [24]. Enhanced bone formation has been shown after injection of mesenchymal stem cell- (MSC-) seeded *β*-TCP in fibrin glue admixtures into the subcutaneous space on rat dorsa [24]. MSCs in fibrin gel used as a cell delivery system* in vivo* migrate out of the gel and invade ceramic scaffold [25].

A comparative study of BCP with different HA/TCP ratios in mandibular bone defects in minipigs showed that BCP20/80 results in similar bone formation as autologous bone after 52 weeks [26]. BCP scaffolds with a HA/*β*-TCP ratio of 60/40, a total porosity of 70% of which 50% were macropores (diameter 300–600 *μ*m) and 30% were micropores (diameter < 10 *μ*m) combined with fibrin sealant stimulated bone formation in animals and humans [20–23]. A pore size of 300–400 *μ*m enhances bone formation and promotes neovascularization which is crucial before osteogenesis can take place in rats [27]. Therefore, in this study BCP biomaterials were used with a porosity of 90%, and a pore size of 500–1000 *μ*m. Human MSCs seeded on BCP60/40 or BCP20/80 incorporated subcutaneously in the back of immunodeficient mice showed the highest amount of bone filling in the pore space and even distribution throughout the entire porous structure of the implant in BCP20/80-loaded composites [28].

Since both osteogenic and vasculogenic differentiation potential of regeneration-competent ASCs seeded on BCP60/40 or BCP20/80 incorporated in fibrin gels is crucial for bone formation in cell-based bone constructs, we aimed to test the osteogenic and/or vasculogenic differentiation potential as well as degradation of composites consisting of human ASCs seeded on BCP60/40 or BCP20/80 incorporated in fibrin gels for bone regeneration. We hypothesized that BCP60/40- and BCP20/80-based composites would enhance osteogenic and vasculogenic differentiation potential of ASCs. We expected that ASCs in BCP20/80-based composites would result in earlier osteogenic differentiation compared to ASCs in BCP60/40-based composites since degradation rate of BCP20/80-based composites is expected to be higher. ASCs were seeded on BCP60/40 or BCP20/80, with similar pore size and porosity, and then incorporated in fibrin gels or directly incorporated in fibrin gels, and cultured for 11 days. Cell attachment to both BCPs was assessed 30 min after cell seeding, and cell proliferation, osteogenic and vasculogenic differentiation potential, and fibrin degradation were assessed up to 11 days of culture.

## 2. Materials and Methods

### 2.1. Biphasic Calcium Phosphate Scaffolds

Two different calcium phosphate scaffolds were used: (1) Straumann® BoneCeramic 60/40 (Institut Straumann AG, Basel, Switzerland), a porous BCP scaffold composed of 60% HA and 40%  *β*-TCP (BCP60/40), and (2) Straumann BoneCeramic 20/80 (Institut Straumann AG, Basel, Switzerland), a porous BCP scaffold composed of 20% HA and 80%  *β*-TCP (BCP20/80). To avoid differences in osteogenic and vasculogenic differentiation potential of ASCs seeded on BCP60/40 or BCP20/80 incorporated in fibrin gels caused by the BCP fabrication process, the two different BCPs were produced by the same company, and had similar pore size and porosity (Table 1).

### 2.2. Donors

Subcutaneous adipose tissue was harvested from residues of abdominal wall resections of five healthy female donors (aged 33, 40, 47, 50, and 54), who underwent elective abdominal wall correction at the Tergooi Hospital Hilversum and a clinic in Bilthoven, Netherlands. The Ethical Review Board of the VU Medical Center, Amsterdam, Netherlands, approved the protocol. Informed consent was obtained from all patients.

### 2.3. Isolation and Culture of Human ASCs

ASCs were isolated from the resection material as described with minor modifications [12]. In brief, adipose tissue was cut into small pieces and enzymatically digested with 0.1% collagenase A (Roche Diagnostics GmbH, Mannheim, Germany) for 45 min at 37°C in phosphate-buffered saline (PBS) containing 1% bovine serum albumin (Roche Diagnostics GmbH) under continuous stirring. Ficoll® density-centrifugation step (Lymphoprep*™*; Axis-Shield, Oslo, Norway; 1000 ×g for 20 min, *ρ* = 1.077 g/mL Ficoll, osmolarity 280 ± 15 mOsm) was performed to remove remaining erythrocytes from the stromal vascular fraction. After centrifugation, the resulting stromal vascular fraction pellet containing ASCs was resuspended in Dulbecco's modified Eagle's medium (Life Technologies*™* Europe BV, Bleiswijk, Netherlands), counted, frozen, and stored in liquid nitrogen until further use. Heterogeneity studies including cell characterization and multipotent differentiation potential of these cells have been reported previously by our group [12].

Cryopreserved stromal vascular fraction-containing cell suspensions of the abovementioned donors were pooled and cultured in *α*-Minimum Essential medium (*α*-MEM; Gibco, Life Technologies, Waltham, MA, USA) with 5% platelet lysate (see below), 100 U/mL penicillin (Sigma-Aldrich, Hamburg, Germany), 100 *μ*g/mL streptomycin sulfate (Sigma-Aldrich), and 10 IU/mL heparin (LEO Pharma A/S, Ballerup, Denmark) to prevent coagulation at 37°C in a humidified atmosphere with 5% CO_2_. The medium was refreshed three times a week. After reaching confluency, cells were harvested by incubation with 0.25% trypsin (Gibco, Invitrogen, Waltham, MA, USA) and 0.1% ethylenediaminetetraacetic acid (Merck, Darmstadt, Germany) in PBS at 37°C, replated, cultured until passage 2 (P2), and stored in liquid nitrogen until further use. Cryopreserved pooled ASCs-containing cell suspensions were thawed and seeded at 0.5 × 10^5^ cells per T-225 culture flask (Greiner Bio-One, Kremsmuenster, Austria) in *α*-MEM with 2% platelet lysate, antibiotics, and 10 IU/mL heparin. Cells were cultured until P3-P4, and used for preparation of composites (see below).

### 2.4. Platelet Lysate

Pooled platelet products from five donors were obtained from the Bloodbank Sanquin (Sanquin, Amsterdam, The Netherlands). Platelet lysate was obtained by lysing the platelets through temperature-shock by freezing at −80°C, thawing, and centrifugation at 600 ×g for 10 min to eliminate remaining platelet fragments. The supernatant was added at 2% (v/v) to the medium.

### 2.5. Fibrin Gel

Human fibrinogen plasminogen-depleted protein (Enzyme Research Laboratories, South Bend, IN, USA) was dissolved in Medium 199 (M199; Gibco, Life Technologies) with antibiotics at 37°C for 1 h. Solubilized fibrinogen was filtered through a 0.2 *μ*m filter (Millipore, Amsterdam, Netherlands), and the concentration measured with a Synergy HT® spectrophotometer (BioTek Instruments Inc., Winooski, VT, USA). To prepare fibrin gel, 2 mg/mL fibrinogen solution was polymerized with 1.0 IU/mL bovine *α*-thrombin (Enzyme Research Laboratories, South Bend, IA, USA) in a buffer containing 50 mM sodium citrate, 0.2 M sodium chloride, and 0.1% polyethylene glycol-8000, for 1 h at room temperature followed by 1 h at 37°C, and used for fibrin coating of polystyrene 48-well culture plates (Cellstar, Greiner Bio-One International GmbH, Frickenhausen, Germany), as well as for preparation of BCP60/40- and BCP20/80-based composites, and ASCs in gels (see below).

### 2.6. Preparation and Culture of Composites

**Figure 1:**
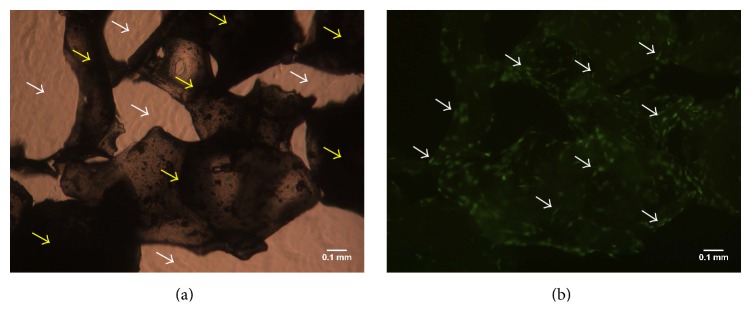
BCP-based composite consisting of ASCs seeded on BCP scaffold incorporated in fibrin gel immediately after preparation. (a) BCP60/40 particles in fibrin gel visualized by light microcopy. Yellow arrows, BCP; white arrows, fibrin gel. (b) ASCs visualized by fluorescence (green) on the same BCP60/40 particles in fibrin gel. White arrows: ASCs. Magnification 40x.

Cultured ASCs were washed three times with PBS to remove platelet lysate, and seeded on either BCP60/40 or BCP20/80 and then incorporated in fibrin gels to prepare BCP60/40- and BCP20/80-based composites, or directly incorporated in fibrin gels. Twenty-five to 30 mg BCP60/40 or BCP20/80 was hydrated in PBS for 30 min. After PBS removal, 1 × 10^5^ ASCs in 100 *μ*L *α*-MEM were allowed to attach for 30 min at room temperature. Unattached cells were counted using a counting chamber (Optik Labor, Lancing, UK). Cell-seeded BCP scaffolds were embedded in fibrin gel and placed on fibrin-coated plates. Immediately after preparation, some composites were used for light microscopy to show the BCP particles in fibrin gel (Figure 1(a)), and to visualize ASCs on these particles in gel by cytotracker green (Invitrogen) according to the manufacturer's instructions (Figure 1(b)). As a control, 1 × 10^5^ ASCs were embedded in fibrin and placed on fibrin-coated plates.

BCP60/40- and BCP20/80-based composites, and ASCs in gels were cultured in *α*-MEM without phenol red (Gibco, Life Technologies, Waltham, MA, USA) with 2% platelet lysate, antibiotics, 50 *μ*g/mL 2-phospho-L-ascorbic acid trisodium salt (Sigma-Aldrich, Steinheim, Germany), and 10 IU/mL heparin for 11 days at 37°C in a humidified atmosphere with 5% CO_2_. Medium was refreshed after 7 days.

### 2.7. Human ASC Proliferation in Composites and Fibrin Gels

Proliferation was assessed by determining cell number in BCP60/40- and BCP20/80-based composites and fibrin gels at days 1 and 11 by using alamarBlue® fluorescent assay (Invitrogen, Frederick, MD, USA), according to the manufacturer's instructions. We found a linear relationship between alamarBlue fluorescence and cell number (data not shown). Fluorescence was read in medium samples at 530 nm with a Synergy HT spectrophotometer.

### 2.8. Alkaline Phosphatase Activity

Alkaline phosphatase (ALP) activity was measured to assess the osteoblastic phenotype of ASCs in BCP60/40- and BCP20/80-based composites and fibrin gels after 1 and 11 days of culture. Both composites and fibrin gels were transferred to 24-well culture plates (Cellstar), washed with PBS, crushed in 300 *μ*L Milli-Q water, and stored at −20°C prior to further use. ALP activity was measured in the cell lysate using 4-nitrophenyl phosphate disodium salt (Merck, Darmstadt, Germany) as a substrate at pH 10.3, according to the method described by Lowry [29]. The absorbance was read at 405 nm with a Synergy HT spectrophotometer. ALP activity was expressed as *μ*M per ng DNA.

### 2.9. Analysis of Gene Expression

At days 1, 7, and 11 of culture, BCP60/40- and BCP20/80-based composites and fibrin gels were transferred to 24-well culture plates, washed with PBS, crushed in 750 *μ*L TRIzol® reagent (Life Technologies, Waltham, MA, USA), and stored at −20°C until further use. Total RNA was isolated using RNeasy® Mini Spin Columns (Qiagen Sciences, Gaithersburg, MD, USA) according to the manufacturer's instructions, and stored at −20°C until further use. Complementary DNA (cDNA) synthesis was performed using SuperScript® VILO*™* cDNA Synthesis kit (Invitrogen, Life Technologies, Carlsbad, CA, USA), with 10.5 *μ*L total RNA in a 15 *μ*L reaction mix containing 3 *μ*L VILO Reaction Mix and 1.5 *μ*L SuperScript Enzyme Mix in a thermocycler GeneAmp® PCR System 9700 PE (Applied Biosystems, Foster City, CA, USA). cDNA was stored at −20°C prior to quantitative real-time PCR (qPCR) analysis, and diluted 5x for gene expression analysis. qPCR reactions were performed using 2 *μ*L cDNA per reaction (10 *μ*L total reaction volume containing 10 pmol of each primer) and LightCycler® 480 SYBR® Green I Mastermix (Roche Diagnostics, Mannheim, Germany) in a LightCycler 480 (Roche Diagnostics). qPCR conditions for all genes were as follows: 10 min preincubation at 95°C, followed by 35 cycles of amplification at 95°C for 2 s, 56°C for 8 s, 72°C for 10 s, and 82°C for 5 s, after which melting curve analysis was performed. With Light Cycler® software (version 1.2), crossing points were assessed and plotted versus the serial dilution of known concentrations of the standard (human primary bone: 2.5–0.004 ng/*μ*L). A human trabecular bone sample (surgical waste) was taken from the femoral head, immediately (within 1 h) after hip surgery for cox-arthrosis, and used as positive control. The protocol was approved by the Ethical Review Board of the VU University Medical Center. PCR efficiency (*E*) was obtained by using the formula *E* = 10^−1/slope^. Data were used only if *E* = 1.85–2.0. For gene expression analysis, the values of target gene expression were normalized to* YWHAZ* housekeeping gene expression to obtain relative gene expression. qPCR was used to assess expression of the following genes:* KI67*, runt-related transcription factor-2 (*RUNX2*), osteonectin (*ON*), dentin matrix acidic phosphoprotein-1 (*DMP1*), collagen-1 (*COL1*), collagen-3 (*COL3*), cluster of differentiation-31 (*CD31*), vascular endothelial growth factor-165 (*VEGF165*), vascular endothelial growth factor-189 (*VEGF189*), and endothlin-1 (*EDN1*). Primer sequences used for qPCR are listed in Table 2.

### 2.10. Fibrin Degradation

Fibrin degradation products were quantified using an enzyme-linked immunosorbent assay as described [30]. Briefly, the antibody fibrin degradation products-14 (FDP-14; TNO, Quality of Life, Leiden, Netherlands) recognizing different epitopes of fibrin degradation products was used as catching antibody. Fibrin degradation product concentrations in the medium of BCP60/40- and BCP20/80-based composites and fibrin gels were investigated after 1, 4, 7, and 11 days of culture, and Biopool standard (Trinity Biotech, Wicklow, Ireland) was used as a reference. Finally, monoclonal antibody D-dimer-13 (DD-13; TNO) labeled with horseradish peroxidase was used as tagging antibody. The coloring reaction was performed using 3,3′,5,5′-tetramethybenzidine (Sigma-Aldrich, St. Louis, MO, USA) and stopped with 1 M H_2_SO_4_. The optical density was read at 450 nm with Synergy HT spectrophotometer.

### 2.11. Statistical Analysis

Data were obtained from quadruple cultures of three independent experiments for BCP60/40-based composites and fibrin gels, and two independent experiments for BCP20/80-based composites. Data are presented as mean ± SEM. Two-tailed unpaired *t*-test was used to compare cell attachment to BCP60/40 and BCP20/80. Differences in alamarBlue fluorescence, ALP activity, and gene expression between BCP60/40- and BCP20/80-based composites and fibrin gels were tested with one-way variance of analysis (ANOVA). To compare fibrin degradation, one-way repeated measures ANOVA was performed. Differences were considered significant if *p* < 0.05. Statistical analysis was performed using IBM® SPSS® Statistics version 21 software package (SPSS Inc., Chicago, IL, USA) and GraphPad Prism® 5.0 (GraphPad Software Inc., La Jolla, CA, USA).

## 3. Results

### 3.1. Cell Attachment to BCP60/40 and BCP20/80

Cell attachment to BCP60/40 and BCP20/80 was similar (BCP60/40: 92730 ± 640, mean cell number ± SEM, *n* = 60 samples; BCP20/80: 98100 ± 350, *n* = 30 samples). After incorporation of cell-seeded BCP in fibrin gel, microscopic observations showed migration of cells towards the fibrin gel in both composites at day 1 (data not shown). The exact number of migrated cells could not be determined with the currently available assays. Future studies have to reveal the precise contribution of cells on BCP and migrated cells to the osteogenic and vasculogenic differentiation potential of both composites.

### 3.2. Increased Cell Proliferation and ALP Activity in BCP60/40- and BCP20/80-Based Composites

**Figure 2:**
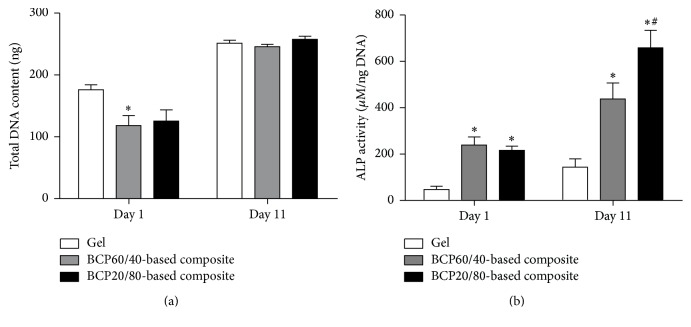
ASC proliferation and osteogenic differentiation in BCP60/40- and BCP20/80-based composites and fibrin gels at day 1 and 11 of culture. (a) Total DNA content in BCP60/40- and BCP20/80-based composites and fibrin gels. The increase in total DNA content from day 1 to day 11 was significantly higher in BCP60/40- and BCP20/80-based composites compared to fibrin gels, indicating similar proliferation in both composites, but increased proliferation in both composites compared to fibrin gels. (b) ALP activity in BCP60/40- and BCP20/80-based composites and fibrin gels. BCP20/80-based composites showed higher ALP activity compared to BCP60/40-based composites at day 11. ALP activity of both BCP60/40- and BCP20/80-based composites was higher compared to fibrin gels at days 1 and 11. Values are mean ± SEM (*n* = 8–12).  ^*∗*^Significant effect of BCP60/40- or BCP20/80-based composites compared to fibrin gels, *p* < 0.05.  ^#^Significantly different from BCP60/40-based composites, *p* < 0.05. BCP, biphasic calcium phosphate; ALP, alkaline phosphatase.

Cell proliferation in BCP60/40- and BCP20/80-based composites was similar, and 1.4–1.5-fold higher compared to fibrin gels (Figure 2(a)). BCP20/80-based composites showed a 1.5-fold increased ALP activity compared to BCP60/40-based composites at day 11 (Figure 2(b)). ALP activity in both composites was higher compared to fibrin gels at days 1 (4.6–5.0-fold) and 11 (3.1–4.6-fold; Figure 2(b)).

### 3.3. Enhanced Osteogenic Differentiation Potential of BCP20/80-Based Composites

**Figure 3:**
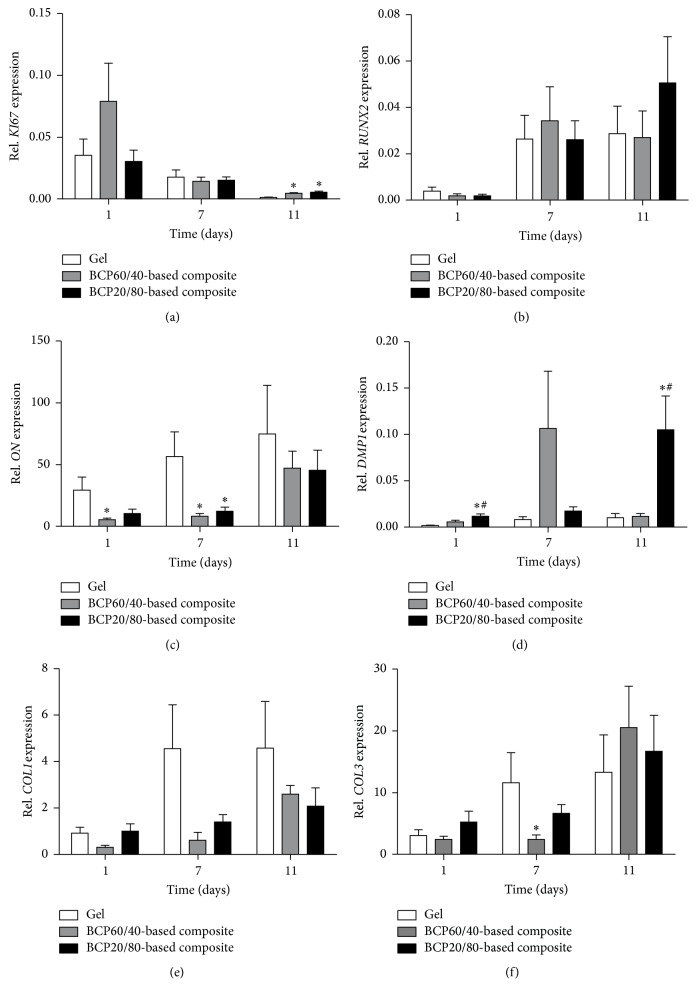
Gene expression of the proliferation marker* KI67* and osteogenic differentiation markers* RUNX2*,* ON*,* DMP1*,* COL1*, and* COL3* in BCP60/40- and BCP20/80-based composites and fibrin gels at days 1, 7, and 11 of culture. (a) BCP60/40- and BCP20/80-based composites showed increased* KI67* gene expression compared to fibrin gels at day 11. (b)* RUNX2* gene expression was similar for BCP60/40- and BCP20/80-based composites and fibrin gels at all time points. (c) BCP60/40- and BCP20/80-based composites showed similar* ON* gene expression at all time points. (d) BCP20/80-based composites showed increased* DMP1* gene expression compared to BCP60/40-based composites, as well as to fibrin gels at days 1 and 11. (e, f) Gene expression of* COL1* and* COL3* in BCP60/40- and BCP20/80-based composites was similar at all time points. Values are mean ± SEM (*n* = 7–12).  ^*∗*^Significant effect of BCP60/40- or BCP20/80-based composites compared to fibrin gels, *p* < 0.05.  ^#^Significantly different from BCP60/40-based composites, *p* < 0.05. BCP, biphasic calcium phosphate;* KI67*, proliferation marker;* RUNX2*, runt-related transcription factor-2;* ON*, osteonectin;* DMP1*, dentin matrix acidic phosphoprotein-1;* COL1*, collagen-1;* COL3*, collagen-3.

No differences were observed in gene expression of the proliferation marker* KI67* in both composites at all time points.* KI67* gene expression was increased in BCP60/40- (3.0-fold) as well as BCP20/80-based composites (4.0-fold) compared to fibrin gels at day 11 (Figure 3(a)). Gene expression of the early osteogenic differentiation marker* RUNX2* in both composites was similar as well as compared to fibrin gels at all time points (Figure 3(b)). Expression of the early-to-late osteogenic differentiation marker* ON* in both composites was also similar at all time points (Figure 3(c)). BCP60/40-based composites resulted in decreased* ON* gene expression compared to fibrin gels at days 1 (0.2-fold) and 7 (0.1-fold).* ON* expression in BCP20/80-based composites was also decreased compared to fibrin gels at day 7 (0.2-fold; Figure 3(c)). Gene expression of the late osteogenic differentiation marker* DMP1* in BCP20/80-based composites was increased compared to BCP60/40-based composites at days 1 (2.1-fold) and 11 (9.2-fold).* DMP1* expression in BCP20/80-based was also increased compared to fibrin gels at days 1 (6.8-fold) and 11 (10.4-fold; Figure 3(d)). The extracellular matrix proteins* COL1* and* COL3* are expressed in osteogenesis and vasculogenesis.* COL1* gene expression was similar in both composites as well as compared to fibrin gels at all time points (Figure 3(e)). BCP60/40- and BCP20/80-based composites showed no significant differences in* COL3* gene expression at all time points (Figure 3(f)).* COL3* gene expression in BCP60/40-based composites was decreased compared to fibrin gels at day 7 (0.2-fold; Figure 3(f)).

### 3.4. Enhanced Vasculogenic Differentiation Potential of BCP20/80-Based Composites

**Figure 4:**
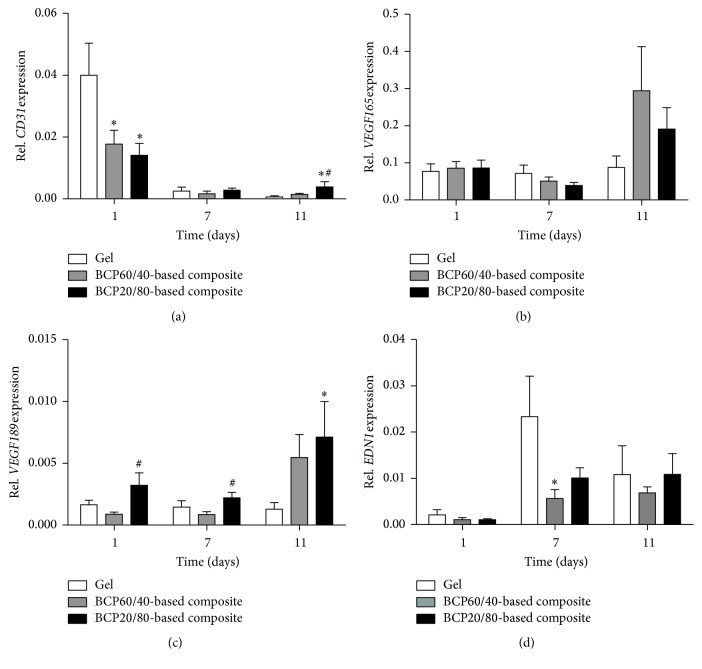
Gene expression of the vasculogenic differentiation markers* CD31*,* VEGF165* and* VEGF189*, and* EDN1* in BCP60/40- and BCP20/80-based composites, and fibrin gels at days 1, 7, and 11 of culture. (a) BCP20/80-based composites showed increased* CD31* gene expression compared to BCP60/40-based composites at day 11, as well as to fibrin gels. (b) BCP60/40- and BCP20/80-based composites demonstrated similar* VEGF165* gene expression at all time points. (c) BCP20/80-based composites showed increased* VEGF189* gene expression compared to BCP60/40-based composites at days 1 and 7. (d) BCP60/40- and BCP20/80-based composites showed similar* EDN1* gene expression at all time points. Values are mean ± SEM (*n* = 5–12).  ^*∗*^Significant effect of BCP60/40- or BCP20/80-based composites compared to fibrin gels, *p* < 0.05.  ^#^Significantly different from BCP60/40-based composites, *p* < 0.05. BCP, biphasic calcium phosphate;* CD31*, cluster of differentiation-31;* VEGF165*, vascular endothelial growth factor-165;* VEGF189*, vascular endothelial growth factor-189;* EDN1*, endothelin-1.

BCP20/80-based composites showed increased* CD31* gene expression compared to BCP60/40-based composites at day 11 (2.8-fold), as well as compared to fibrin gels (6.2-fold; Figure 4(a)).* CD31* gene expression in both composites was decreased (2.3–2.8-fold) compared to fibrin gels at day 1 (Figure 4(a)). Gene expression of* VEGF165* in both composites was similar, as well as compared to fibrin gels at all time points (Figure 4(b)). BCP20/80-based composites showed increased* VEGF189* gene expression at days 1 (3.6-fold) and 7 (2.6-fold) compared to BCP60/40-based composites, as well as compared to fibrin gels at day 11 (5.5-fold; Figure 4(c)). Gene expression of* EDN1* in both composites showed no significant differences at all time points (Figure 4(d)). BCP60/40-based composites resulted in decreased* EDN1* gene expression compared to fibrin gels at day 7 (0.2-fold; Figure 4(d)).

### 3.5. Differences in Fibrin Degradation between Composites

**Figure 5:**
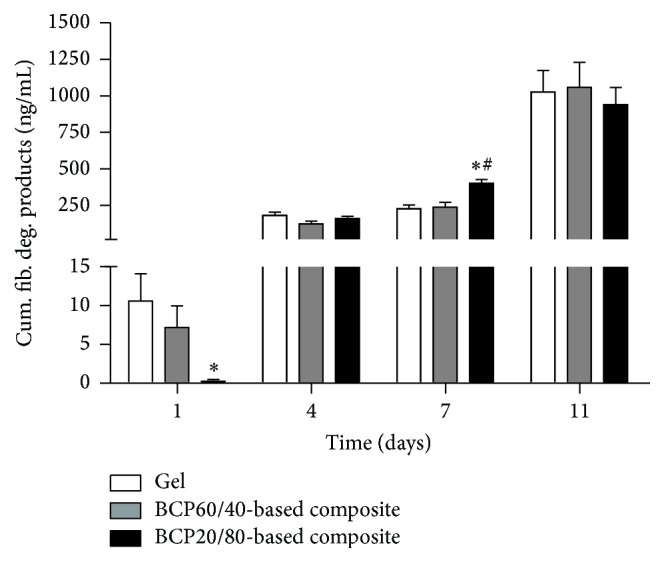
Differences in fibrin degradation of BCP60/40- and BCP20/80-based composites and fibrin gels. BCP60/40- and BCP20/80-based composites degradation was similar at day 11. BCP20/80-based composites resulted in increased concentration of fibrin degradation products compared to BCP60/40-based composites at day 7 of culture, as well as to fibrin gels. Values are mean ± SEM (*n* = 8–12).  ^*∗*^Significant effect of BCP60/40- or BCP20/80-based composites compared to fibrin gels, *p* < 0.05.  ^#^Significantly different from BCP60/40-based composites, *p* < 0.05. BCP, biphasic calcium phosphate; cum, cumulative; fib, fibrin; deg, degradation.

A decreased concentration of fibrin degradation products in the medium of BCP20/80-based composites compared to fibrin gels was observed at day 1 (0.02-fold; Figure 5). The degradation products concentration was increased in BCP20/80-based composites compared to BCP60/40-based composites (1.7-fold) and fibrin gels at day 7 (1.8-fold), but reached similar levels at day 11.

## 4. Discussion

This study aimed to test the osteogenic and/or vasculogenic differentiation potential as well as degradation of composites consisting of human ASCs seeded on BCP60/40 or BCP20/80 incorporated in fibrin gels for bone regeneration. We found that (i) ASC attachment to both BCPs was similar; (ii) proliferation in BCP60/40- and BCP20/80-based composites was similar, but higher compared to fibrin gels; (iii) BCP20/80-based composites showed higher ALP activity compared to BCP60/40-based composites; (iv) gene expression of the late osteogenic marker* DMP1* in BCP20/80-based composites was increased compared to BCP60/40-based composites as well as to fibrin gels; (v) higher gene expression of the vasculogenic markers* CD31* and* VEGF189* was seen in BCP20/80-based composites compared to both BCP60/40-based composites and fibrin gels. Therefore, our results showed enhanced osteogenic and vasculogenic differentiation potential in BCP20/80-based composites compared to BCP60/40-based composites* in vitro*, suggesting that BCP20/80-based composites might be more promising for* in vivo* bone augmentation than BCP60/40-based composites.

We found that ASC attachment to both BCPs was similar, as shown earlier [31], indicating that different HA/*β*-TCP ratios of BCP did not affect ASC attachment. To ensure cell survival, proliferation, and differentiation of transplanted cell-seeded scaffolds after implantation, adequate nutrient and oxygen supply is crucial. A composite will easily become hypoxic after implantation in the body. ASCs cultured on fibrin gel-coated plates show enhanced proliferation under severe hypoxic conditions (1% oxygen) compared to conventional oxygen conditions (20% oxygen) [32]. Therefore, enhanced proliferation in both composites might be explained by a stimulatory effect of the BCP scaffold [33], and/or a hypoxic microenvironment, which likely also occurs after* in vivo* implantation.

The concept that degradation by-products can influence stem cell function, including cell fate decisions, is emerging [34]. TCP supports cell ingrowth and promotes osteogenic differentiation of osteoprogenitor cells [35]. The extent of dissolution of BCP depends on the HA/*β*-TCP ratio; the lower the ratio, the more the dissolution [36]. We found higher ALP activity, indicating enhanced osteogenic differentiation in BCP20/80-based composites compared to BCP60/40-based composites. This can be explained by the low HA/*β*-TCP ratio in BCP20/80 compared to BCP60/40, indicating higher Ca^2+^ ion release in BCP20/80-based composites which is crucial for bone formation. Degradation of surrounding material, that is, fibrin gel, can lead to cell-traction forces, which are crucial for osteogenic differentiation of human MSCs in a three-dimensional context and might explain the acceleration in osteogenic differentiation in both composites and fibrin gels [37]. This speculation is consistent with our fibrin degradation products data showing increased fibrin degradation in BCP20/80-based composites compared to BCP60/40-based composites at day 7. We found higher expression of the late stage osteogenic differentiation marker* DMP1*, but similar expression of early and early-to-late osteogenic differentiation markers in BCP20/80-based composites compared to BCP60/40-based composites, suggesting that BCP20/80-based composites were in a later stage of osteogenic commitment compared to BCP60/40-based composites as well as to fibrin gels. Therefore, BCP20/80-based composites seem promising for implantation* in vivo* for enhanced bone formation.

Vascular development needs to be induced prior to osteogenesis. CD31 is expressed on the cell surface of endothelial and hematopoietic cells. BCP20/80-based composites showed increased* CD31* expression compared to BCP60/40-based composites indicating higher vessel-forming potency. During culture,* CD31* expression decreased, while* VEGF165*,* VEGF189*, and* EDN1* expression increased over time in both composites. The high expression of these vasculogenic genes indicates increased vasculogenic differentiation potential of both composites, although* CD31* expression decreased, which suggests that the number of endothelial progenitor cells had decreased over time. VEGF probably functions as a hypoxia-inducible angiogenic factor [38]. We found increased expression of the vasculogenic marker* VEGF189* in BCP20/80-based composites compared to fibrin gels. Therefore, BCP20/80-based composites might offer a hypoxic microenvironment for the ASCs, resulting in increased VEGF expression. Endothelin-1 is a potent vasoconstrictor and has been identified originally in vascular endothelial cells. In osteoblasts, it stimulates inorganic phosphate transport, which is important for bone matrix calcification [39].* EDN1* expression was increased during culture in both BCP60/40- and BCP20/80-based composites, which indicates enhanced vasculogenic and osteogenic differentiation potential. Future studies are needed to verify possible differences in blood vessel and bone formation using composites with ASCs, BCPs with different HA/*β*-TCP ratios, and fibrin gels* in vivo*.

The stem cell-matrix interface is a complex, dynamic microenvironment in which the cell and the material cooperatively dictate one another's fate and regulate stem cell differentiation [37]. Changes in fibrin composition will create different matrix stiffness and architectural properties, which will have impact on cellular response. Stem cells are extremely sensitive to elasticity of their surrounding matrix, through mechanosensitive ion channels, focal adhesions, cell surface receptors, actin cytoskeleton, and cell-cell adhesions, and they respond dramatically in lineage to the matrix presented [40]. Understanding the mechanisms of cellular sensory capabilities of ASCs will be relevant for application of our composites in tissue engineering. Fibrin remodelling was increased in BCP20/80-based composites compared to BCP60/40-based composites, but reached a similar level at the end of culture. The difference in fibrin remodelling possibly results in differences in cellular behaviour. The increase in fibrin degradation products during culture suggests dissolution of fibrin. After implantation of BCP60/40- and BCP20/80-based composites in the body, dissolution of the three-dimensional biological matrix fibrin will occur. As in fracture healing, cells migrate through the three-dimensional biological matrix and might be directly or indirectly directed through cytokines or growth factors released [41]. We observed migration of ASCs from BCP into the fibrin gel, which likely also occurs after implantation of composites* in vivo*. Later, fibrous tissue will be formed replacing fibrin, thereby holding cell-seeded BCP in place. Thus, our composites seem promising candidates for bone augmentation* in vivo*.

BCP-based composites might fit in a one-step surgical procedure. We used an expanded stem cell pool consisting of a homogeneous mixture of cells. However, only a freshly isolated stromal vascular fraction will fit in a one-step surgical procedure [42]. Stromal vascular fraction consists of a heterogeneous mixture of cells including endothelial cells and lineage-committed progenitor cells and is not characterized while our stem cell pool was characterized [14, 43]. To determine whether freshly isolated stromal vascular fraction gives similar results in BCP-fibrin composites, it is necessary to test the osteogenic and/or vasculogenic differentiation potential of composites with freshly isolated stromal vascular fraction seeded on BCPs with different HA/*β*-TCP ratios incorporated in fibrin gels.

**Figure 6:**
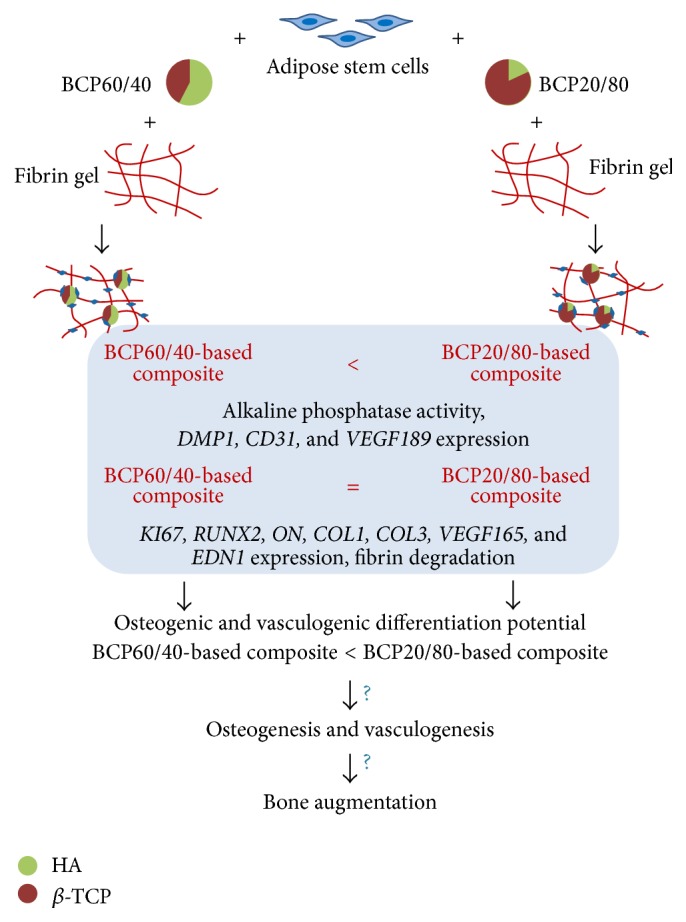
Development of BCP60/40-based composites and BCP20/80-based composites. Human adipose stem cells were seeded on BCP60/40 or BCP20/80 and incorporated in fibrin gels. BCP20/80-based composites showed enhanced osteogenic differentiation and vasculogenic potential compared to BCP60/40-based composites* in vitro*. =, similar in both composites; <, higher in BCP20/80-based composites compared to BCP60/40-based composites; HA, hydroxyapatite; *β*-TCP, *β*-tricalcium phosphate;* KI67*, proliferation marker;* RUNX2*, runt-related transcription factor-2;* ON*, osteonectin;* DMP1*, dentin matrix acidic phosphoprotein-1;* COL1*, collagen-1;* COL3*, collagen-3;* CD31*, cluster of differentiation-31;* VEGF165*, vascular endothelial growth factor-165;* VEGF189*, vascular endothelial growth factor-189;* EDN1*, endothelin-1.

In summary, BCP20/80-based composites showed increased ALP activity as well as* DMP1*,* CD31*, and* VEGF189* gene expression compared to BCP60/40-based composites (Figure 6). In addition, BCP20/80-based composites showed increased fibrin degradation. Therefore, we conclude that BCP20/80-based composites showed enhanced osteogenic and vasculogenic differentiation potential compared to BCP60/40-based composites* in vitro*, suggesting that BCP20/80-based composites might be more promising for* in vivo* bone augmentation than BCP60/40-based composites.

## Figures and Tables

**Table 1:** Characteristics of the different BCPs used. Composition, particle size, porosity, and pore width of Straumann BoneCeramic 60/40 and Straumann BoneCeramic 20/80. HA: hydroxyapatite; *β*-TCP: *β*-tricalcium phosphate; BCP: biphasic calcium phosphate; SSA: specific surface area. Magnification 50x and 5000x.

Scaffold	Composition	Particle size (*µ*m)	Crystal size (*µ*m)	Porosity (%)	Interconnected pores (*µ*m)	SSA (10^−3^ m^2^/g)	Microporosity (%)
Straumann	60% HA	500–1000	0.6–6.0	90	100–500	6.9	2.0
BoneCeramic	40% *β*-TCP						
BCP60/40							
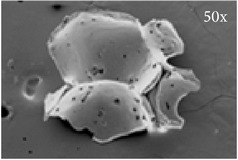	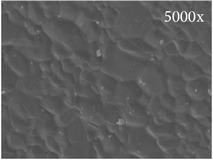						
Straumann	20% HA	500–1000	1.0–6.0	90	100–500	9.5	2.0
BoneCeramic	80% *β*-TCP						
BCP20/80							
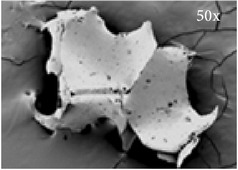	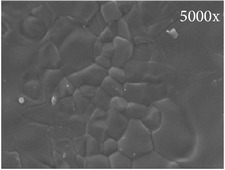						

**Table 2:** Primer sequences for determination of osteogenic and vasculogenic differentiation potential through PCR. *YWHAZ*: tyrosine 3-monooxygenase/tryptophan 5-monooxygenase activation protein, zeta; *KI67*: proliferation marker; *RUNX2*: runt-related transcription factor-2; *ON*: osteonectin; *DMP1*: dentin matrix acidic phosphoprotein-1; *COL1*: collagen-1; *COL3*: collagen-3;* CD31*: cluster of differentiation-31;* VEGF165*: vascular endothelial growth factor-165; *VEGF189*: vascular endothelial growth factor-189; *EDN1*: endothlin-1; bp: base pairs.

Target gene		Oligonucleotide sequence	Product size (bp)	Annealing temperature (°C)
*YWHAZ*	Forward	5′ gATgAAgCCATTgCTGAACTTg 3′	229	56
	Reverse	5′ CTATTTgTgggACAgCATggA 3′		

*KI67*	Forward	5′ CCCTCAgCAAgCCTgAgAA 3′	202	56
	Reverse	5′ AgAggCgTATTAggAggCAAg 3′		

*RUNX2*	Forward	5′ ATgCTTCATTCgCCTCAC 3′	156	56
	Reverse	5′ ACTgCTTgCAgCCTTAAAT 3′		

*ON*	Forward	5′ CTgTCCAggTggAAgTAgg 3′	233	56
	Reverse	5′ gTggCAggAAgAgTCgAAg 3′		

*DMP1*	Forward	5′ TAggCTAgCTggTggCTTCT 3′	375	56
	Reverse	5′ AACTCggAgCCgTCTCCAT 3′		

*COL1*	Forward	5′ TCCggCTCCTgCTCCTCTTA 3′	336	56
	Reverse	5′ ggCCAgTgTCTCCCTTg 3′		

*COL3*	Forward	5′ gATCCgTTCTCTgCgATgAC 3′	279	56
	Reverse	5′ AgTTCTgAggACCAgTAggg 3′		

*CD31*	Forward	5′ AACAggAgggAgAgTATTACTg 3′	236	56
	Reverse	5′ TggTACTgCTggCCTggA 3′		

*VEGF165*	Forward	5′ ATCTTCAAgCCATCCTgTgTgC 3′	224	56
	Reverse	5′ CAAggCCCACAgggATTTTC 3′		

*VEGF189*	Forward	5′ ATCTTCAAgCCATCCTgTgTgC 3′	289	56
	Reverse	5′ CACAgggAACgCTCCAggAC 3′		

*EDN1*	Forward	5′ gTTTgTggCTTgCCAAggA 3′	207	56
	Reverse	5′ ACgTgCTCgggAgTgTTgA 3′		
